# VA-ECOM assisted percutaneous mechanical thrombectomy treatment high-risk pulmonary embolism

**DOI:** 10.3389/fcvm.2024.1457157

**Published:** 2024-12-24

**Authors:** Zhenhang Zhou, Yaoyang Zhong, Jianbo Hu, Zhonghua Wu, Liping Zou, Zhihe Deng, Guoshan Bi, Xin Shen, Xianpeng Dai, Zhijia Huang, Guozuo Xiong, Yiming Xu, Liming Deng

**Affiliations:** ^1^ Department of Vascular Surgery, The Second Affiliated Hospital, Hengyang Medical School, University of South China, Hengyang, Hunan, China; ^2^ Hunan Province Thrombotic Disease Prevention and Treatment Clinical Medical Research Center, Hengyang, Hunan, China; ^3^ Hunan Provincial Key Clinical Specialty, Hengyang, Hunan, China; ^4^ Department of Critical Care Medicine, The Second Affiliated Hospital, Hengyang Medical School, University of South China, Hengyang, Hunan, China; ^5^ Department of Vascular Surgery, Zhongshan Hospital Xiamen University, Xiamen, Fujian, China

**Keywords:** pulmonary embolism, cardiogenic shock, VA-ECOM, percutaneous mechanical, prognosis

## Abstract

**Background:**

Percutaneous mechanical thrombectomy (PMT) is increasingly used in the treatment of intermediate and high-risk acute pulmonary embolism (PE), and the treatment of high-risk PE with the aid of veno-arterial extracorporeal membrane oxygenation (VA-ECMO) has also been reported. However, there are few reports of VA-ECOM-assisted PMT in the treatment of high-risk PE. The purpose of this study is to summarize the data of 11 patients with high-risk PE treated with VA-ECMO assisted PMT, and propose feasible treatment methods for such patients.

**Methods:**

This multicenter retrospective study included patients with acute high-risk PE who were treated with VA-ECMO-assisted PMT from January 2021 to June 2024. The analysis focused on the right/left ventricle ratio, biomarkers, and pulmonary artery pressure before and after the VA-ECMO-assisted PMT treatment.

**Results:**

All 11 high-risk PE patients suffered cardiac arrest before treatment, computered tomograhy pulmonary angiography (CTPA) confirmed the diagnosis of PE, and all patients received VA-ECMO-assisted PMT therapy. The median age of the 11 patients was 54 years (range 18–72), the median duration of ECMO was 4.48 days (range 1.04–18.02), and the mean hospitalization time was 21 days (range 14–112). All patients received percutaneous thrombectomy, achieving a 100% technical success rate. The mortality rate was 27.3% during the 90-day follow-up. The 12-month mortality rate was 36.4%.

**Conclusion:**

VA-ECMO-assisted PMT technology can rapidly improve pulmonary hemodynamics while maintaining stable blood flow, thereby reducing in-hospital mortality in high-risk patients with pulmonary embolism complicated by cardiac arrest.

## Introduction

Pulmonary embolism (PE) is one of the leading causes of death from cardiovascular disease, with an annual incidence of 39-115 per 100,000 people and approximately 300,000 deaths per year in the United States (1). Acute high-risk PE is characterized primarily by right ventricular (RV) dysfunction, hypoxemia, hemodynamic instability, and cardiac arrest (2), which has an incidence rate of 5%, and it is associated with 30-day mortality rate ranging from 16% to 46% for patients in shock and approaching 52% to 84% for those with cardiac arrest (1). The purpose of treatment of high-risk PE is to rapidly reduce thrombotic load, immediately improve hemodynamic status and reduce the occurrence of thromboembolic pulmonary hypertension. Current guidelines recommend systemic thrombolysis as the first-line treatment for high-risk PE patients (2), However, it was less effective in patients with cardiac arrest (1).

Veno-arterial extracorporeal membrane oxygenation (VA-ECMO) improves hemodynamic status by bypassing the RV, restoring circulation, reducing RV overload, and restoring tissue oxygenation. Thus, in the case of acute PE, VA-ECMO can serve as a bridge to recovery or treatment. Multiple studies have reported significant benefits from temporary use of mechanical circulatory support via VA-ECMO as a bridging therapy during resection with medication or mechanical thrombolysis or embolization (3–6). Among patients with acute PE cardiac arrest, the short-term survival rate of patients treated with VA-ECMO is 34% (7), while that of patients not treated with VA-ECMO is 8.5%–18.3% (8, 9).

Recent studies have demonstrated the feasibility, safety, and effectiveness of percutaneous mechanical thrombectomy (PMT) for moderate risk PE (10, 11). Studies have also reported the efficacy of interventional therapy and VA-ECMO support in patients with acute high-risk PE and thrombolysis contraindications (5). Currently, the use and outcomes of VA-ECMO alone or in combination with mechanical or pharmaceutical thrombolysis or embolectomy are poorly reported. Therefore, the purpose of this study was to investigate the efficacy of VA-ECMO assisted percutaneous mechanical thrombectomy in patients with acute PE.

## Patients and methods

The study was based on data from two cohorts of PE from January 2021 to June 2024. The two clinical cohorts included were: The Second Affiliated Hospital of University of South China and Zhongshan Hospital Xiamen University. This study was performed according to the Declaration of Helsinki, and ethical approval was obtained from the Institutional Ethics Committees of The Second Affiliated Hospital of University of South China and Zhongshan Hospital Xiamen University. The study participants' clinicopathological information was reviewed with their written informed consent.

**Figure 1:**
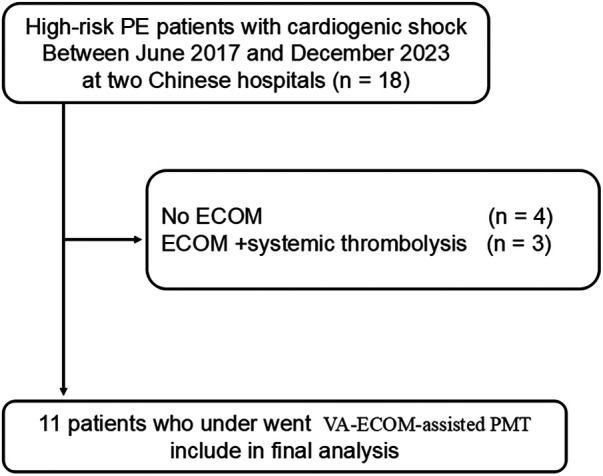
The flow chart depicts the total number of participants ultimately included in this study.

All patients enrolled were computered tomograhy pulmonary angiography (CTPA) confirmed to have PE. High-risk PE was defined as PE with at least one of the following factors: cardiac arrest, obstructive shock, or persistent systemic hypotension. Right ventricle dysfunction was defined as an elevated RV: LV ratio (>0.9). VA-ECMO inclusion criteria were as follows: (1) Preoperative diagnosis of PE by CTPA, substantial thrombotic burden in the pulmonary artery and/or bedside echocardiography showing evidence of pulmonary hypertension impacting RV function, and presence of hypotension and shock; (2) In patients with reversible cardiac arrest, VA-ECMO is performed and PE is determined by CT imaging after VA-ECMO intubation. The study flow diagram is illustrated in Figure 1. All 11 patients experienced cardiac arrest and underwent cardiopulmonary resuscitation. After resuscitation, their hemodynamics remained unstable, so VA-ECMO support was provided. Considering the relative contraindications for thrombolytic therapy and the patients' unstable conditions, the PERTs team decided to proceed with endovascular thrombectomy after thorough discussion.

### PMT with VA-ECMO support procedure

To achieve hemodynamic and respiratory stabilization, the patient first underwent femoro-femora VA-ECMO. Percutaneous access to the femoral artery and vein was achieved, and peripheral VA-ECMO was initiated. The heparinized VA-ECMO blood flow was maintained at 4.5 L/min. Subsequently, pulmonary angiography was performed, revealing pulmonary artery thrombosis. Mechanical thrombofragmentation and aspiration were then carried out. Thrombectomies were performed using the Acostram peripheral to your suction system (Beijing Senruida Medical Technology Co., LTD., China). First, the catheter tip was positioned on the thrombus, and the aspiration-retraction device was activated to generate suction. Based on angiographic visualization of the thrombus location, the catheter tip position was continuously adjusted during the procedure to maximize thrombus extraction. Angiography was used to evaluate the effectiveness of the thrombectomy and the improvement in hemodynamics. After stabilizing the patient's hemodynamics, VA-ECMO was removed, and the patient continued on low molecular weight heparin to prevent recurrence of thromboembolism.

### Technical success

Technical success was defined as the removal of the majority of the thrombus and the recovery of the majority of pulmonary artery perfusion. Hemodynamic assessment was performed by measuring pulmonary artery (PA) pressure before and immediately after surgery. A reduction in PA pressure was also indicators of the technique's success. Technical failure is defined as no clot removal or clinical deterioration after surgery.

#### Collection of baseline characteristics

Patient basic demographics and clinical information were obtained from the hospital's database and analyzed retrospectively. The data encompassed age, gender, body mass index (BMI), simplified Pulmonary Embolism Severity Index (sPESI), and cardiovascular risk elements such as hypertension, smoking habits, and past medical history. Initial presenting symptoms included dyspnea, chest pain, syncope, and cardiac arrest. Imaging findings covered right ventricular (RV) dilatation, the RV to left ventricular (LV) ratio, and the presence of saddle embolism. Laboratory tests highlighted the significance of high-sensitive troponin T, Brain Natriuretic Peptide (BNP), and lactate levels. Furthermore, the study examined the association with VA-ECMO, focusing on the duration of VA-ECMO support, complications related to VA-ECMO, and the time of intensive care unit (ICU) stay.

### Study outcomes

The primary endpoint was 90-day mortality. Secondary endpoint was 12-month mortality and RV recovery. After hospital discharge, follow-up was performed up to 3 months by telephone contact with the patients and their family. Complications were recorded in patients receiving ECMO, including the occurrence of stroke, the need for renal replacement therapy, catheter site infection, ventilator-associated pneumonia, sepsis.

### Statistical analysis

All analyses were performed using SPSS (SPSS® software, version 22.0, SPSS Inc., Chicago, Illinois). Continuous variables were expressed as mean ± standard deviation or median (interquartile range). Categorical variables were expressed in terms of frequency (percentage). Student's *t* test was used to compare groups when a continuous variable showed normal distribution; otherwise, the Mann–Whitney *U* test was used. The Kaplan–Meier method and log-rank test were used to estimate overall survival. A *p*-values <0.05 was considered statistically significant.

## Results

From January 2021 to June 2024, two hospitals participating in this study identified a total of 18 cases of high-risk PE. Of these, 4 patients did not receive VA-ECMO support. Regarding treatment, 3 patients were administered VA-ECMO in conjunction with systemic thrombolysis, while another 11 received percutaneous thrombectomy with the assistance of VA-ECMO (Figure 1). Among the patients undergoing thrombectomy, there was an equal distribution of 6 males and 5 females, with an average age of 54 years (range 18 to 72). Detailed baseline characteristics of these patients are provided in Table 1.

**Table 1:** Baseline patient characteristic.

Demographics	*n* or median (% or range)
Total number of patients *(n*, %)	11 (100%)
Median age (years, range)	54 (18–72)
Male sex, (*n*, %)	6 (54.5%)
Body mass index (kg/m^2^), (median, range)	23.15 (21.4–24.8)
Hypertension (*n*, %)	1 (9.1%)
Diabetes mellitus (*n*, %)	2 (18.2%)
Hyperlipidemia (*n*, %)	3 (27.3%)
Malignancy (*n*, %)	1 (9.1%)
Chronic kidney disease (*n*, %)	1 (9.1%)
VTE history (*n*, %)	2 (25%)
Active smoking (*n*, %)	3 (27.3%)
Recent trauma (*n*, %)	4 (36.4%)
Recent surgery (*n*, %)	5 (45.5%)
Recent stroke (*n*, %)	1 (12.5%)
sPESI (median, range)	2 (1–3)
COVID-19, (*n*, %)	0 (0%)
CPR (*n*, %)	11 (100%)
Initial symptoms	
Dyspnea (*n*, %)	11 (100%)
Chest pain (*n*, %)	11 (100%)
Syncope (*n*, %)	11 (100%)
Cardiac arrest (*n*, %)	11 (100%)
Imaging	
CTPA (*n*, %)	11 (100%)
RV dilatation (*n*, %)	11 (100%)
RV/LV ratio (median, range)	1.96 (1.63–2.56)
Saddle embolism (*n*, %)	4 (36.4%)
Echocardiography (*n*, %)	11 (100%)
Laboratory	
High-sensitive troponin T positive, *n* (%)	11 (100%)
BNP (ng/ml)	2,221 (137–5,466)
Lactate (mmol/L)	6.2 (3.5–20)
VA-ECMO correlation	
VA-ECMO (*n*, %)	11 (100%)
VA-ECMO duration (days, range)	4.48 (1.04–18.02)
Complication of VA-ECOM (*n*, %)	1 (9.1%)
PMT (*n*, %)	11 (100%)
90-day Mortality (*n*, %)	3 (27.3%)
12-month Mortality (*n*, %)	4 (36.4%)
ICU time (days, range)	19 (5–89)
Hospitalization time (days, range)	21 (14–112)

VTE, venous thromboembolish; CPR, cardiopulmonary resuscitation; CTPA, computed tomography pulmonary angiography; RV, right ventricle; LV, left ventricle; BNP, brain natriuretic peptide; VA-ECMO, veno-arterial extracorporeal membranous oxygenation; ICU, intensive care unit; PMT, percutaneous mechanical thrombectomy.

All patients exhibited symptoms of dyspnea (100%) and syncope (100%). The median sPESI was 2 (range 1–3). Every patient experienced cardiac arrest either before admission or during their hospital stay, necessitating pharmacological vasopressor support. Radiologically, CT scans revealed right ventricular (RV) dilation in all cases, with a median RV/LV ratio of 1.96 (range 1.63–2.56). Saddle embolism was observed in 36.4% of the patients, and echocardiographic evaluation was performed preoperatively for all individuals. Laboratory tests indicated a median lactate level of 6.2 mmol/L (range 3.5–20), a median BNP level of 2221 ng/ml (range 137–5,466), and elevated levels of high-sensitive troponin T across the board (Table 1).

**Figure 2:**
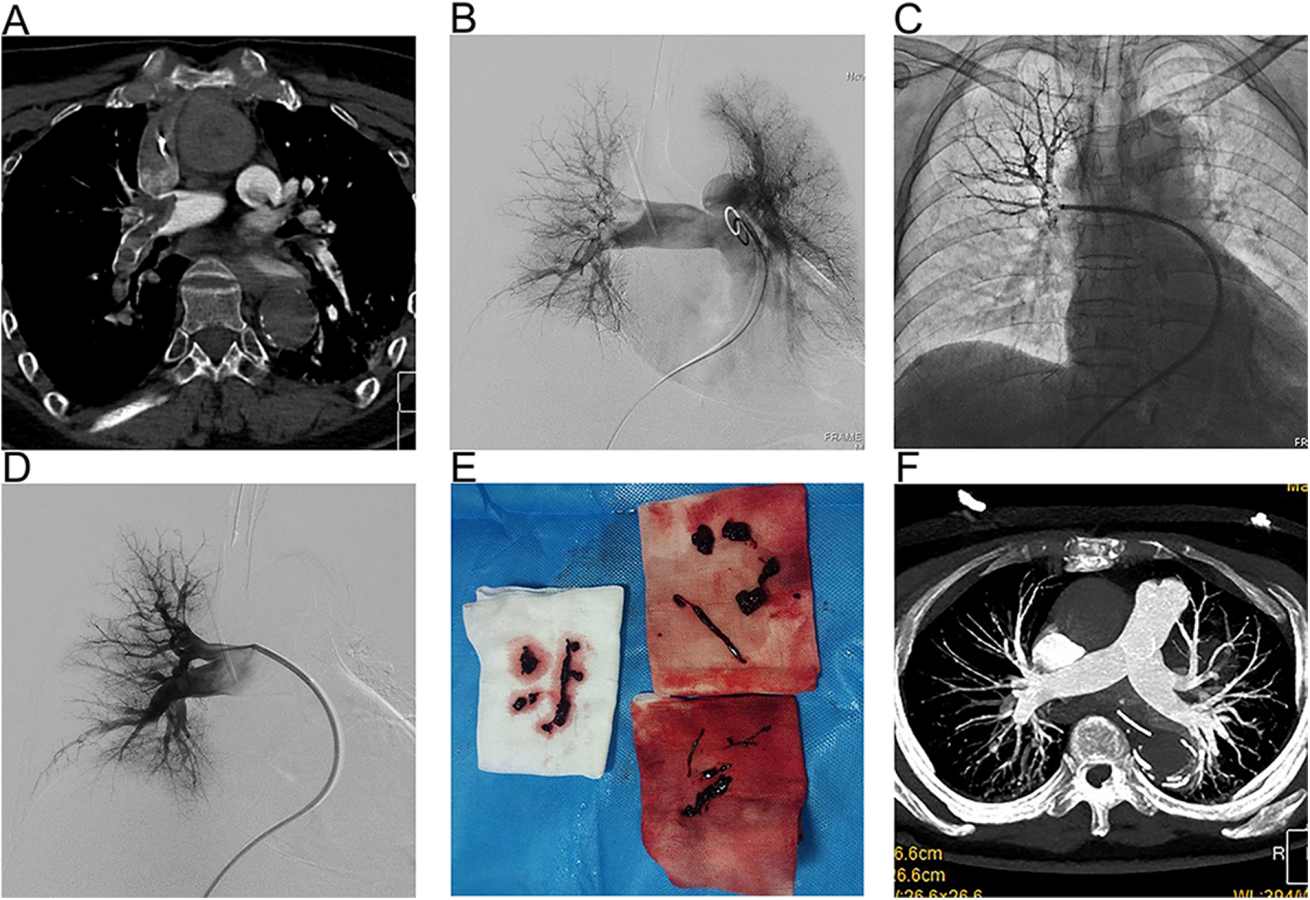
A representative case of VA-ECOM-assisted PMT for PE. **(A)** CTA revealed a large pulmonary embolism and occlusion of the pulmonary artery; **(B)** Digital subtraction pulmonary angiograms before thrombectomy shown significant right pulmonary artery obstruction; **(C)** Application of bolting system; **(D)**) Angiograms after thrombectomy demonstrated pulmonary artery markedly improved perfusion; **(E)** The thrombus was obtained during thrombectomy; **(F)** Follow-up CTA demonstrating no thrombus in the pulmonary artery.

All patients started treatment within 24 h of developing PE, each presenting with at least one absolute or relative contraindication to thrombolytic therapy, including recent trauma, stroke, and surgery. Among them, 11 patients underwent cardiopulmonary resuscitation (CPR) prior to their surgeries, with VA-ECMO support being immediately initiated upon successful resuscitation. All 11 of these patients received percutaneous thrombectomy, achieving a 100% technical success rate, with none requiring conversion to open thrombectomy. Figure 2 presents a case where percutaneous thrombectomy successfully treated a PE. Under VA-ECMO support, the patient's PE was diagnosed through CTPA (Figure 2A), with typical pulmonary angiography images taken before and after the thrombectomy (Figures 2B-D), and the extracted thrombus (Figure 2E). CTPA examination 1 month after surgery showed that the thrombus in pulmonary artery basically disappeared (Figure 2F).

**Figure 3:**
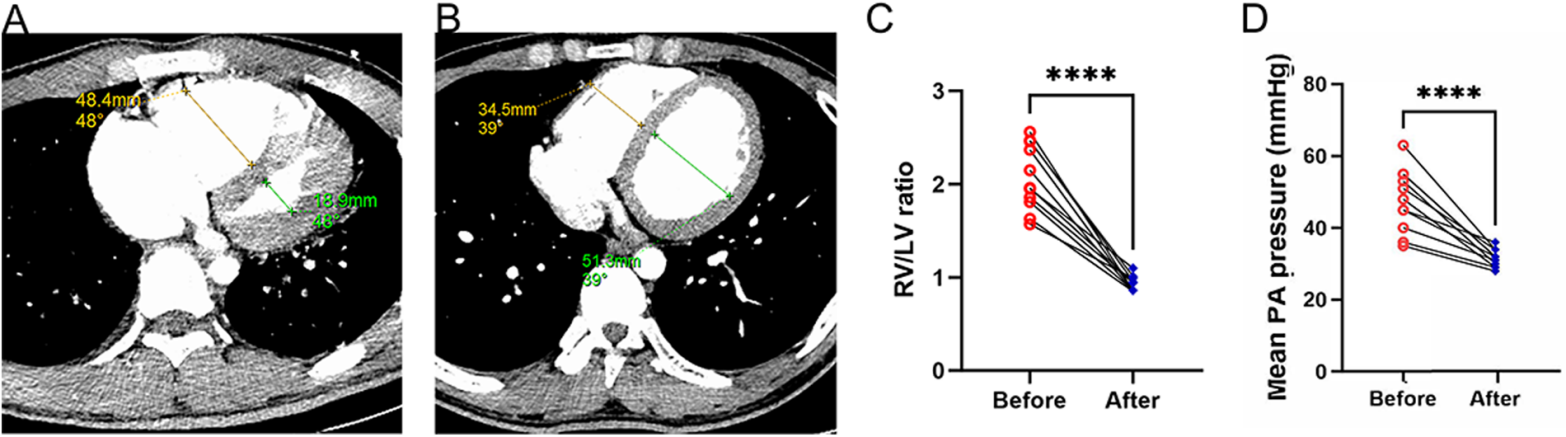
Alterations in right and left ventricular ratios before and after surgery. **(A)** The ratio of the right ventricle to the left ventricle as determined by CT scans prior to thrombectomy; **(B)** The ratio of the right ventricle to the left ventricle as determined by CT scans following thrombectomy; **(C)** Variations in the RV/LV ratio pre- and post-surgery; **(D)** Mean PA pressure was compared before and immediately post PMT.

**Figure 4:**
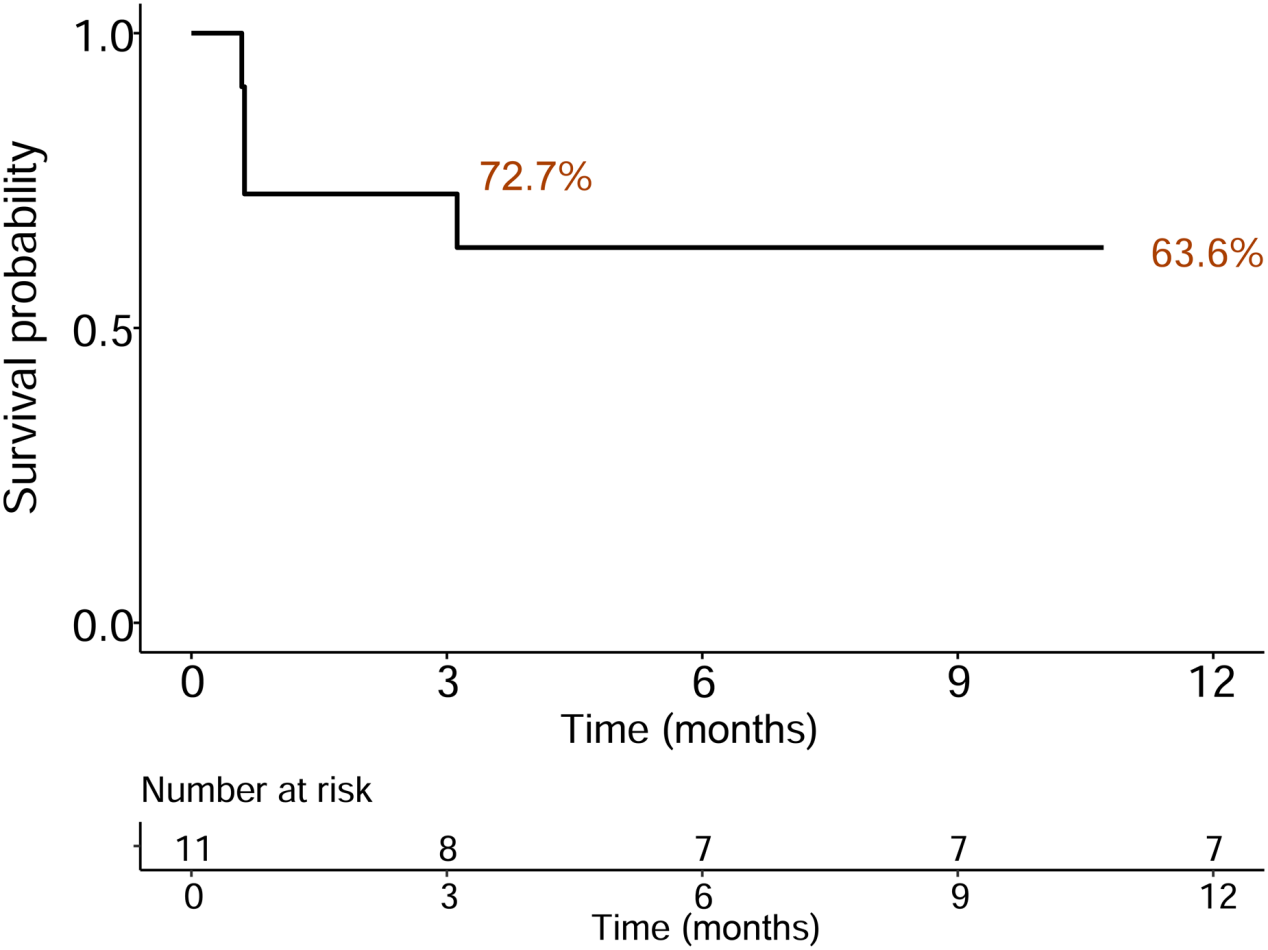
Kaplan–Meier survival curve indicating all-cause mortality following discharge for an average follow-up of 12 months.

Preoperative and postoperative CTPA of 11 patients showed a significant reduction in the ratio of RV/LV ((Figure 3A–C). PA pressure was measured intraoperatively and immediately postoperatively, showing a significant decrease (Figure 3D), with about 70% of patients not needing vasopressor drugs to maintain blood pressure on the day after surgery. Following aspiration thrombectomy, the median time to wean off VA-ECMO was 4.48 days (range 1.04–18.02), while the median stay in the Intensive Care Unit (ICU) was 19 days (range 5–89 days), and the median total hospital stay was 21 days (range 14–112), Only one patient with ECOM-related complications had ventilator-associated pneumonia (Table 1). 19 days post-surgery, one patient died from multi-organ failure, and the other two patients were considered hypoxic encephalopathy, and the patients' families abandoned treatment; another died on day 112 after surgery due to ischemic hypoxic encephalopathy caused by prolonged resuscitation before the initiation of VA-ECMO, resulting in a vegetative state, with the family ultimately deciding to cease further medical intervention. All patients completed a one-year follow-up, with no instances of pulmonary embolism recurrence observed during this period. The study group's all-cause mortality rate was 27.3% within 90 days and reached 36.4% within 12 months (Figure 4).

## Discussion

This represents the inaugural multi-center investigation into the utilization of VA-ECMO-assisted percutaneous mechanical thrombectomy for patients at high risk of pulmonary embolism (PE). The findings demonstrate a marked improvement in hemodynamic parameters among this patient cohort as a result of the surgical intervention. The employment of VA-ECMO for lung support ensured the safety of the procedure, leading to a high rate of surgical success. Additionally, patient mortality rates were notably low, with 72.7% of the patients being successfully discharged and surviving beyond 90 days post-hospitalization. This methodology not only enhances resource efficiency but also significantly improves patient outcomes.

For high-risk PE patients, systemic thrombolytic therapy is generally the preferred treatment. However, it carries a significant risk of severe bleeding complications, with rates reaching up to 8% (12). In such scenarios, VA-ECMO emerges as a crucial intervention, mitigating insufficient perfusion to peripheral organs and reducing the strain on the right ventricle, thus providing essential support for high-risk PE patients (13, 14). Endorsement from the European Society of Cardiology for employing VA-ECMO in high-risk PE cases further solidifies its importance (2). Despite the concerning 30-day mortality rate of 76% observed in high-risk patients receiving thrombolytic therapy with VA-ECMO support (4), the application of VA-ECMO-supported thrombectomy post-acute PE cardiac arrest has shown promising outcomes in select instances for those contraindicated for thrombolysis (15–19). The current study showed that adding systemic thrombolysis did not have significant survival or other benefits in high-risk PE patients receiving VA-ECMO (20). The reasons were considered that mechanical obstruction could not be quickly resolved and hemodynamic support could not be restored. PMT assisted by ECMO can quickly resolve mechanical obstruction and restore blood perfusion to the lung in a short time. Our research bolsters the evidence that VA-ECMO-assisted percutaneous mechanical thrombectomy for high-risk PE not only promptly stabilizes patients and delivers hemodynamic support during the procedure but also significantly improves thrombus clearance through aspiration. This leads to a reduction in pulmonary artery pressure and an enhancement in the right-to-left ventricular ratio, rapidly improving the hemodynamic condition and substantially boosting patient survival chances.

For individuals resuscitated from cardiac arrest due to high-risk PE, thrombolytic therapy is categorically contraindicated. In such scenarios, VA-ECMO and interventional thrombectomy emerge as viable options for patients who cannot undergo systemic thrombolysis. While VA-ECMO alone as a method of reperfusion is an alternative, its safety and effectiveness remain subjects of ongoing debate. Should interventional thrombectomy not yield improvements in hemodynamic stability, surgical thrombectomy may be necessitated, associated with a mortality rate of 19.8% (21). Our application of VA-ECMO-supported percutaneous mechanical thrombectomy in high-risk PE cases achieved a 90-day all-cause mortality rate of 27.3% and 12 months all-cause mortality rate of 36.4%, reflecting greater safety and reduced trauma compared to conventional surgical interventions. Within our study group, the success rate of operation was 100%, although one patient died 19 days after surgery due to multiple organ failure, and the other two patients were considered hypoxic encephalopathy, and the patients' families abandoned treatment, another died 112 days after surgery due to ischemic hypoxic encephalopathy caused by prolonged resuscitation prior to initiation of VA-ECMO. The patient remained in a vegetative state after surgery, leading to the family's decision to discontinue further medical intervention. Notably, no significant bleeding incidents or access-site related hemorrhages were recorded in our patient cohort. However, 8 patients received packed red blood cell transfusions, and hemoglobin levels at discharge were lower than at admission, likely due to blood loss associated with VA-ECMO use. Consequently, VA-ECMO-assisted percutaneous mechanical thrombectomy presents itself as a potentially pivotal interventional strategy in the management of high-risk PE.

The primary limitations of this study are its retrospective nature, the small size of the study population, and its non-randomized approach. Selection bias is present due to the focus on high-risk PE patients who underwent CPR. Owing to the high risk of bleeding with intravenous thrombolysis, only patients who underwent VA-ECMO combined with PMT were selected, excluding a contemporaneous comparison of the safety and efficacy between VA-ECMO combined with PMT and VA-ECMO-assisted thrombolysis. This limitation restricts the ability to extrapolate the results to the general population. Therefore, further prospective and randomized studies are essential to verify these preliminary observations.

## Conclusion

VA-ECMO-assisted PMT technology can rapidly improve pulmonary hemodynamics while maintaining stable blood flow, thereby reducing in-hospital mortality in high-risk patients with pulmonary embolism complicated by cardiac arrest.

## Data Availability

The original contributions presented in the study are included in the article/Supplementary Material, further inquiries can be directed to the corresponding author/s.
